# Multimodal objective assessment of impulsivity in healthy and mood disorder participants

**DOI:** 10.1038/s44277-025-00026-z

**Published:** 2025-02-14

**Authors:** Bishal Lamichhane, Nidal Moukaddam, Ramiro Salas, Wayne Goodman, Ashutosh Sabharwal

**Affiliations:** 1 Electrical and Computer Engineering, Rice University, Houston, TX USA; 2 Menninger Department of Psychiatry, Baylor College of Medicine, Houston, TX USA; 3 Center for Translational Research on Inflammatory Diseases, Michael E DeBakey VA Medical Center, Houston, TX USA; 4 The Menninger Clinic, Houston, TX USA

**Keywords:** Risk factors, Human behaviour

## Abstract

Impulsivity represents an individual’s tendency to act on urges without sufficient forethought. Heightened impulsivity is a hallmark of many mental health disorders. Objective impulsivity assessments could improve risk evaluation, diagnosis, and behavioral outcome monitoring in impulsivity-related health disorders. Towards objective impulsivity assessment, in this work, we identify impulsivity correlates in objective measurements, investigate their complementarity, and contrast impulsivity mechanisms across health conditions. We analyzed behavioral tests, heart rate variability (HRV), and fMRI-based brain connectivity in 227 healthy participants and 34 participants with mood disorders. Impulsivity dimensions had complementary correlates in objective measurements, with fMRI providing the strongest correlates. Multimodal assessment provided high r-squared (adjusted) values in modeling impulsivity of the mood disorder participants (e.g., r-squared of 0.73, *p* < 0.001 for attentional impulsivity) but low r-squared for healthy participants (the best r-squared being 0.17, *p* < 0.001 for sensation seeking impulsivity). The differing association between impulsivity dimensions across the two populations likely indicates a health condition-specific impulsivity mechanism across populations. The complementary nature of objective impulsivity correlates across populations demonstrates the distributed signature of multidimensional impulsivity, likely capturing the complexity of behavioral modeling.

## Lay Summary

Impulsivity is commonly implicated in many mental health disorders. We examined behavioral tests, heart rate variability, and brain connectivity (via fMRI) as objective methods to assess impulsivity in 227 healthy individuals and 34 individuals with mood disorders. fMRI provided the strongest correlates of impulsivity, with multimodal assessment providing the most accurate models of impulsivity across health conditions. Our work lays the ground for future studies to develop robust impulsivity assessments across different populations.

## Introduction

Impulsivity represents an individual’s predisposition to act rapidly on urges without sufficient premeditation or regard for consequences [1]. Health disorders such as substance use disorder (SUD) [1], pathological gambling [2], binge eating disorder [3], and bipolar disorder [4] associates with poor impulse control. Even healthy population could exhibit high impulsivity (poor impulse control), putting these individuals at high risk for health disorders in future [5–7]. Quantitative impulsivity assessments could help in diagnosis, treatment monitoring, and interventions for multiple health disorders. Currently, the predominant tool to assess impulsivity is self-report questionnaires. Since impulsivity is well known to be a multidimensional construct [8, 9], impulsivity questionnaires try to capture the different facets of impulsive behavior. Barratt Impulsiveness Scale (BIS) [10], for example, is a 30-item questionnaire that assesses attentional, motor, and nonplanning impulsivity as different impulsivity dimensions. UPPS-P [11] is another 59-item questionnaire assessing five impulsivity dimensions: negative urgency, lack of premeditation, lack of perseverance, sensation seeking, and positive urgency. The subjective impulsivity assessment using questionnaires could be complemented by objective measurements to improve the reliability of assessments and facilitate frequent passive monitoring with sensor-based measurements. Monitoring the impulsivity of at-risk individuals would allow better behavioral outcome monitoring and timely interventions.

For objective impulsivity assessments, “body signals” involved in the pathways of an impulsive behavior could be utilized. From an information processing perspective, a behavior, including impulsive ones, entails perception (evoking emotions), cognition, premeditation, and action [12]. External or internal stimuli are perceived and internalized (evoking emotions), cognitive premeditation about action choices and execution of the chosen action occurs, and feedback from the outcome is obtained. The emotion processing and response control in impulsive/non-impulsive behaviors implicates particular brain regions such as prefrontal cortex [13], anterior cingulate cortex (ACC) [14, 15], motor cortex [16], and hippocampus [17]. Thus, functional magnetic resonance imaging (fMRI) could reveal functional connections of brain regions implicated in impulsive behaviors to provide neurobiological correlates of impulsivity [18–20]. The neuro-visceral integration model implicates cardiac physiology also in executive functions and inhibitory control [21]. Cardiac physiology is also associated with emotional responses [22] and autonomic regulation linked to impulsivity [23]. Thus, heart rate variability (HRV) could provide physiological correlates of impulsivity [21]. Apart from fMRI and HRV, behavioral tests designed to prime impulsive responses from participants could provide behavioral correlates of impulsivity for objective impulsivity assessment [24].

Previous studies have investigated how impulsivity could be represented in objective measurements, evaluating several modalities spanning behavioral, physiological, and neurobiological measurements [25–29]. However, a multimodal investigation has not been conducted, creating gaps in knowledge of objective impulsivity assessment. The contribution of various objective measurements to model impulsivity, their hierarchy, and possible complementarity needs to be clarified. Measurement modalities providing impulsivity correlates vary in their ease of use and cost. Thus, knowing the best impulsivity correlates in various application settings, e.g., for a behavioral follow-up, is not feasible with current knowledge. A multimodal regression model of impulsivity was pursued in a recent work [30] using mobile sensing features obtained from 26 healthy participants. Mobile sensing features provided behavioral correlates of impulsivity. However, physiological and neurobiological correlates could be the missing components to better model impulsivity - the developed model could explain only 1 to 30% of the variance in BIS-based impulsivity across participants. The possibility of reliably modeling impulsivity using objective measurements remains an open question.

In this work, we investigated multimodal modeling of impulsivity using behavioral, physiological, and neurobiological measurements. We conducted a study with 34 mood disorder participants, primarily with major depressive disorder or bipolar disorder (referred to as the MIND-2 study/dataset). Behavioral tests, HRV, and resting-state fMRI-based brain connectivity were obtained from the participants. Different modalities were complementary in representing impulsivity and jointly produced an r-squared (adjusted) as high as 0.73 (*p* < 0.001) for attentional impulsivity. Our work is the first to combine multiple objective measurement modalities to obtain a highly accurate representation of impulsivity in mood disorder participants. We also analyzed the relation of objective measurements with impulsivity in a healthy population (no significant physical or mental health ailments) of 227 participants in the LEMON dataset [31]. The behavioral, physiological, and neurobiological correlates were complementary in modeling impulsivity in the LEMON dataset as well. However, the r-squared for the modeling was lower than those obtained in the MIND-2 dataset. For example, the best r-squared obtained was only 0.17 (*p* < 0.001) for sensation seeking impulsivity. The differences in impulsivity modeling results and the differing associations between impulsivity dimensions across the MIND-2 and LEMON dataset demonstrates the varying impulsivity mechanisms across health conditions.

## Method

### Study group

We used datasets from two studies in this work. The first study is a new clinical study, MIND-2 study, with 34 participants with mood disorders. The second is the LEMON study with an open-source dataset [31] from 227 healthy participants. The details of both studies are provided below.

#### MIND-2 study

We conducted the MIND-2 study at Baylor College of Medicine and Harris Health System (IRB approval identifier H-44164). The study enrolled 34 participants. We used the Mini International Neuropsychiatric Interview (MINI) [32] to characterize the participant’s clinical condition and obtain their mental health diagnosis. Most of the participants were diagnosed with major depressive disorder (MDD) (N = 21) or bipolar disorder (N = 11), possibly representing different impulsivity levels since bipolar disorder is associated with higher impulsivity [33]. The other two participants were diagnosed with post-traumatic stress disorder and panic disorder. Of the participants, 15 were male, and the rest were female. The average age of the participants was 27.3 ± 5.2 years (minimum: 18, maximum: 35 years). We enrolled participants from a limited age group to control age-related impulsivity differences. The participants’ demographics are also summarized in Table S1 of the Supplementary. All participants belonged to Houston, one of the most diverse large cities in the USA. Ten participants were Hispanic, and twenty-four participants were non-Hispanic. Among the Hispanic participants, five identified as white. For the non-Hispanic participants, the composition was thirteen whites, eight African-American/black, and three Asian. Among the twenty-eight participants who provided information about their education, two had no high school-level education, seven had high school-level education, thirteen joined college but did not earn a degree, one had an associated degree, and five had an undergraduate degree or higher. Of the twenty-nine participants who disclosed their relationship status, four were married or in a relationship, and twenty-five were not in a relationship. The Barratt’s Impulsivity Scale (BIS) [10] and UPPS-P Scale [11] (negative urgency, lack of premeditation, lack of perseverance, sensation seeking, positive urgency) were used to obtain questionnaire-based impulsivity of the participants. BIS is a 30-item questionnaire producing impulsivity scores that characterize three subscales for second-order factors. The subscales are attentional, motor, and nonplanning impulsivity [10]. UPPS-P, on the other hand, depicts five impulsivity dimensions with a 59-item questionnaire: negative urgency, lack of premeditation, lack of perseverance, sensation seeking, and positive urgency [11].

#### LEMON study

The LEMON study [31] enrolled 227 participants, with 82 female/145 male participants and an average age of 39.2 +/−20.3 years. The participants were recruited cross-sectionally at Leipzig, Germany. One hundred seventy-six participants reported having a high school degree on the academic track (gymnasium), 49 with a high school degree on the vocational track (Realschule or hauptschule), one with no high school degree, and one did not report their education level. Of the 218 participants who provided information about their relationship status, 149 indicated they were in a relationship while the remaining 69 were not. Exclusion criteria for the participants included any significant ailments or health issues such as cardiovascular disease, neurological disorder, history of psychiatric illness requiring hospitalization, substance use, and history of malignant disease. All participants were screened with semi-structured telephone interviews and a physician assessment to ensure they did not meet the exclusion criteria. Thus, the LEMON dataset could be considered a healthy participant cohort dataset. The LEMON study used the UPPS questionnaire to assess participant’s impulsivity. UPPS differs from the UPPS-P used in our MIND-2 study in missing only the fifth impulsivity dimension of positive urgency.

### Common objective measurements in both datasets

Objective behavioral, physiological, and neurobiological measurements were available in the MIND-2 and LEMON datasets. These measurements could be employed to obtain impulsivity correlates and are briefly described below.

#### Behavioral measurements

In the MIND-2 study, we used the computerized IMT/DMT [24] and arrow-based Flanker tests [34, 35]. Error rate and response time were computed as likely impulsivity correlates [35]. From the LEMON dataset, we used the TAP-I assessment (test of attentional performance – incompatibility) results [36]. The test error rate and response time were evaluated as possible correlates of impulsivity.

#### Physiological measurements

In the MIND-2 study, the HRV of the participants was calculated using the PPG signal (Photoplethysmography) obtained from the pulse oximeter in the PulseCam system [37]. The PulseCam measurements were obtained with the participants under stress since stress reveals impulsivity differences between individuals [38]. We used the Math task (MT) and the speech task (ST) from the Trier Social Stress Test [39] to induce stress. Each stress task lasted for about five minutes. The PPG signal from the pulse oximeter was processed using the biopeaks toolbox [40] to detect the systolic peaks. To account for possible errors in peak detection owing to the noise and artifact in the PPG signal, we visually inspected all detected peaks and corrected for any incorrectly located peaks using the biopeaks graphical user interface. Based on the systolic peak locations in the PPG signal, the following HRV features across the stress task phase were computed: (i) mean heart rate, (ii) RMSSD (root mean square of the sum of successive differences), (iii) SDRR (standard deviation of the peak-peak intervals), (iv) pNN-50 (percentage of peak-to-peak intervals lower than 50 ms), (v) pNN-20 (percentage of peak-to-peak intervals lower than 20 ms) (vi) SD1, SD2, and SD1/SD2 features from the R-R interval Poincare plot (where SD1 and SD2 represent the major and minor axis of the fitted ellipse to the R-R interval Poincare plot). For the physiological measurements in the LEMON study, we also used the PPG signal obtained from the participants and computed HRV features as possible impulsivity correlates. The same set of HRV features was calculated as in the MIND-2 dataset.

#### Neurobiological measurements

We obtained fMRI scans of the participants during the resting and task states (while completing the behavioral tests) in the MIND-2 study. The resting-state fMRI of the participants was also available in the LEMON dataset and was used to obtain neurobiological correlates of impulsivity. We refer to Supplementary Section S3 for details about the fMRI scan parameters in these two datasets and fMRI data quality analysis.

To identify the correlates of impulsivity in brain connectivity, we pursued seed-to-ROI (region of interest) connectivity analysis [41]. The anterior cingulate cortex (ACC) region of the brain was selected as the seed. ACC, positioned between the prefrontal cortex and the limbic system which are involved in emotion regulation and cognition, plays a role in psychopathology [42], impulse control [43], conflict processing [44], decision-making [45], as well as intermediate and long-term action regulation [46]. The ROI was defined by the Harvard-Oxford atlas’s cortical & subcortical areas and AAL atlas’s cerebellar areas, as used in the CONN toolbox [47] (hereby referred to as the CONN atlas). The functional connectivity from ACC to other ROI regions in the CONN atlas was computed for both the MIND-2 dataset and the LEMON dataset. The association of these connectivities with impulsivity was assessed to identify the neurobiological correlates of impulsivity.

The ROI defined by the CONN atlas represents fixed brain regions. Impulsivity could implicate brain regions that pervade ROI boundaries or be localized in only a smaller portion of a larger ROI. To identify localized brain regions whose connectivity to ACC is associated with impulsivity, we pursued seed-based connectivity (SBC) analysis [41]. Such localization of brain regions implicated in impulsivity could be especially relevant in future studies to identify intervention targets in clinical populations, e.g., for neuromodulation-based impulsivity management [48–50]. Thus, we pursued SBC analysis in the MIND-2 dataset to identify brain regions implicated in impulsivity for the population with mental health disorders.

### Multimodal regression and prediction model

To evaluate multimodal impulsivity assessment models utilizing the behavioral, physiological, and neurobiological measurements in the MIND-2 and the LEMON datasets, we used a similar methodology and evaluation metric as the previous work on multimodal impulsivity modeling [30]. In particular, regression modeling was used to assess the capabilities of the objective modalities to explain the variability in the target impulsivity measures and prediction models were developed to evaluate the capabilities of the objective modalities to predict the impulsivity of unseen participants. Regression models of impulsivity were evaluated with impulsivity correlates from the three modalities (behavioral, physiological, and neurobiological). We used the ordinary least square regression with feature selection [30] for regression models. Correlation-based feature selection was used to select the top two features per modality to be included in the regression model. As described earlier, the feature pool comprised the error rates, response times from behavioral tests, HRV features from PPG signal, and ACC-based connectivity. The adjusted r-squared metric penalized increasing features in the multimodal regression model. For the prediction model, we used a linear support vector machine evaluated with leave-one-participant-out cross-validation. The regularization parameter *C* in the linear support vector machine was set to the default of the scikit-learn library in Python (*C* = 1.0). The prediction model was evaluated with the correlation between the predicted and reported impulsivity levels as the evaluation metric. We have shared the multimodal regression and prediction modeling for the LEMON dataset as an example at https://github.com/lbishal/multimodal_impulsivity.

## Results

### Relation between impulsivity dimensions

We analyzed the association between the BIS and UPPS-P impulsivity dimensions within the MIND-2 dataset and between the UPPS impulsivity dimensions (an earlier version of UPPS-P with missing fifth impulsivity dimension of positive urgency) within the LEMON dataset. The internal consistency of the subscales for the BIS and UPPS-P/UPPS, quantified with Cronbach’s alpha, were good. The Cronbach’s alpha ranged from 0.73 to 0.91 for BIS and 0.77 to 0.96 for UPPS-P in the MIND-2 dataset. The Cronbach’s alpha ranged from 0.69 to 0.85 for the UPPS in the LEMON dataset [31]. The correlation between the impulsivity dimensions is shown in Supplementary Fig. S1. The impulsivity dimensions showed a strong significant correlation in the MIND-2 dataset. Each dimension had a significant positive correlation with multiple other dimensions except sensation seeking, which was associated with only two other impulsivity dimensions, positive and negative urgency. Compared to the MIND-2 dataset, the observed association between impulsivity dimensions was weaker in the LEMON dataset (average and maximum of absolute correlation coefficient being 0.15 and 0.25 in the LEMON dataset compared to the average and maximum of 0.54 and 0.83, respectively, in the MIND-2 dataset). The inter-dimension associations were different across the dataset. For example, sensation seeking was strongly associated with the lack of premeditation in the LEMON dataset. However, sensation seeking was only associated with urgency (both positive and negative) in the MIND-2 dataset.

The UPPS impulsivity dimensions were assessed for the participants in both the MIND-2 and LEMON datasets. Both datasets demonstrated a widespread impulsivity score among participants (Supplementary Fig. S2). The MIND-2 dataset of mood disorder participants had higher average negative urgency scores than healthy participants in the LEMON dataset.

### Correlates of impulsivity in objective measurements

We computed the correlation between features from objective measurements (fMRI, HRV, and behavioral tests) and impulsivity dimensions in the MIND-2 and LEMON datasets. Full correlation tables for the significant association are provided in the supplementary material (Supplementary Tables S2–S4). Table 1 gives a summary of the obtained correlates. Correlates were defined as features with Pearson’s correlation coefficient of significance value *p* < 0.10 with target impulsivity dimension. A weaker criterion defined correlates since some borderline non-significant correlations could still be helpful for downstream regression and prediction tasks. Correlates of impulsivity dimensions were found in all modalities, i.e., behavioral, physiological (HRV), and neurobiological (fMRI), for the MIND-2 dataset and the LEMON datasets.

**Table 1:** Correlates of impulsivity in different objective measurement modalities.

Dataset	Impulsivity Dimension	Correlates
MIND-2	*BIS*	
	Attentional	HRV, fMRI
	Motor	Behavioral, HRV, fMRI
	Non-planning	Behavioral, HRV, fMRI
	*UPPS-P*	
	Negative Urgency	Behavioral, HRV
	Lack of Premeditation	Behavioral, HRV, fMRI
	Lack of Perseverance	Behavioral, HRV
	Sensation Seeking	-
	Positive Urgency	Behavioral, HRV
LEMON	*UPPS*	
	Negative Urgency	HRV
	Lack of Premeditation	HRV
	Lack of Perseverance	Behavioral, HRV
	Sensation Seeking	Behavioral, HRV, fMRI

IMT/DMT and Flanker test were used as behavioral tests in the MIND-2 dataset, and TAP-I was used as the behavioral test in the LEMON dataset. Heart rate variability (HRV) calculated from PPG signal and functional connectivity from ACC (anterior cingulate cortex), as possible impulsivity correlates, were available in both datasets. Full correlation tables with correlation coefficient and associated significance for all significance values *p* < 0.10 are given in the supplementary material (Supplementary Table S2–S4).

In the MIND-2 dataset, the Flanker error rate had a significant positive correlation with the nonplanning and lack of premeditation. The correlation coefficients were 0.41 (*p* < 0.05) and 0.47 (*p* < 0.05), respectively. The IMT/DMT test provided correlates of complementary impulsivity dimensions, e.g., for motor impulsivity (correlation coefficient of 0.49, *p* < 0.05). In the LEMON dataset, the TAP-I response time had a significant negative correlation with the lack of perseverance and sensation seeking. Higher impulsivity is associated with higher errors and lower response time in behavioral tests. Thus, error rates were positively correlated, while the response time was negatively correlated with impulsivity, as expected. In the physiological modality, several HRV features provided correlates of attentional impulsivity with the SD1 and SD2 features from the HRV Poincare plot having a correlation coefficient as high as 0.53 (*p* < 0.001) with the attentional impulsivity. Among the HRV features with significant association with impulsivity, only the mean heart rate and SD1/SD2 features were negatively associated with impulsivity, while all other HRV features were positively associated. The HRV features provided correlate for all impulsivity dimensions except for the sensation seeking in the MIND-2 dataset and the LEMON datasets. Scatterplots for some example associations between impulsivity and behavioral/physiological features are shown in Supplementary Fig. S4.

#### Seed-based functional brain connectivity at rest and impulsivity

**Fig. 1:**
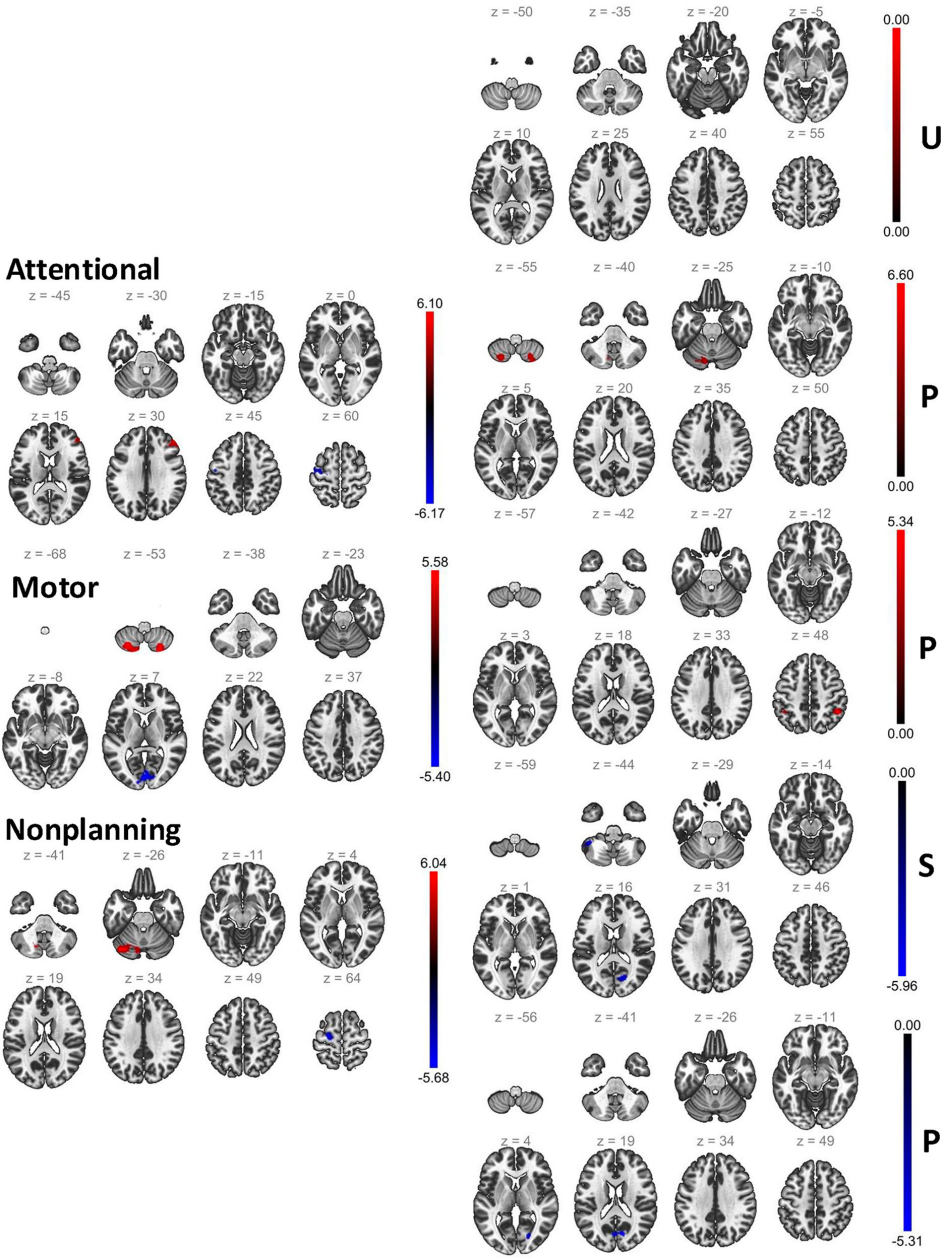
Brain regions implicated in seed-based connectivity analysis from ACC as the seed for the MIND-2 dataset. The left pane shows the results for the BIS dimensions, and the right pane shows the results for the UPPS-P dimensions (U - negative urgency, P - lack of premeditation, P - lack of perseverance, S - sensation seeking, P - positive urgency). For each impulsivity dimension, the slices show the brain region as best visualized in different depths and the t-statistics for the connectivity-impulsivity association is shown by the overlaid color. Different impulsivity dimensions implicated different brain regions, but some common brain regions, such as the left precentral gyrus, cerebellar brain regions, frontal pole, and intracalcarine cortex regions, were commonly implicated across impulsivity dimensions.

We pursued seed-based connectivity with Anterior Cingulate Cortex (ACC) as seed to localize brain regions implicated in impulsivity in the clinical population represented by the MIND-2 dataset (see Methods). ACC is positioned between the prefrontal cortex and subcortical limbic systems (both involved in emotion regulation and cognition) and is commonly implicated in psychopathology [42], impulse control [43], and decision-making [45]. The results are shown in Fig. 1 and summarized in Table 2. The connectivity of ACC to the left precentral gyrus, cerebellar brain regions, frontal pole, and intracalcarine cortex regions was associated with impulsivity dimensions.

**Table 2:** Brain regions associated with impulsivity in the seed-based connectivity from the anterior cingulate cortex (ACC), the brain region linking the prefrontal cortex and limbic systems, in the MIND-2 dataset.

Total					
		Cluster		Peak	
Impulsivity	Main Region	Size	pFDR	Location	T-values
Attentional	Left Precentral Gyrus (−)	749	*<0.001*	−24, −18, +74	−5.77
	Right Frontal Pole (+)	367	*<0.01*	+48, +40, +26	5.60
	Right Intracalcarine Cortex (−)	546	*<0.001*	+04, −76, +02	−5.73
Motor	Left Cerebelum 8 (+)	389	*<0.01*	−28, −68, −54	5.93
	Right Cerebelum 8 (+)	346	*<0.01*	+26, −68, −58	6.02
	Left Precentral Gyrus (−)	302	*<0.01*	−20, −06, +76	−5.81
Nonplanning	Left Cerebelum Crus1 (+)	575	*<0.001*	−38, −74, −28	6.92
	Left Precentral Gyrus (−)	432	*<0.001*	−20, −18, +72	−6.23
Negative					
Urgency	−	−	−		
Lack of	Left Cerebelum Crus1 (+)	837	*<0.001*	−22, −70, −48	7.36
Premeditation	Right Cerebelum 8 (+)	256	*<0.05*	+24, −66, −52	5.40
Lack of	Right Supramarginal Gyrus (+)	269	*<0.01*	+46, −50, +60	4.98
Perseverance	Left Superior Parietal Lobule (+)	210	*<0.05*	−44, −42, +56	5.21
Sensation	Left Cerebelum Crus1 (−)	240	*<0.05*	−48, −56, −36	−6.10
Seeking	Right Intracalcarine Cortex (−)	209	*<0.05*	+18, −68, +14	−4.91
Positive		171	*<0.001*	+12, −72, +18	−5.14
Urgency	Right Intracalcarine Cortex (−)				

All impulsivity dimensions except the negative urgency implicated some brain regions for their significant association with impulsivity. To be noted, the association shown in Table 1 considered ACC - ROI (region of interest from CONN atlas) connectivity, while the analysis presented in this table concerns seed-based connectivity. A voxel-level threshold of *p* < 0.001 uncorrected height threshold on t-statistics of each voxel is used to identify voxels to be retained. Significant clusters of the retained voxels using an 18-connectivity neighborhood with *pFDR* < 0.05 are then identified (parametric analysis based on the Random Field Theory [47]). The main region represented in the significant cluster, total cluster size, peak location within the cluster in the MNI coordinates, and the t-statistics for the connectivity in the cluster are also reported in the table.

### Multimodal regression and prediction model

**Fig. 2:**
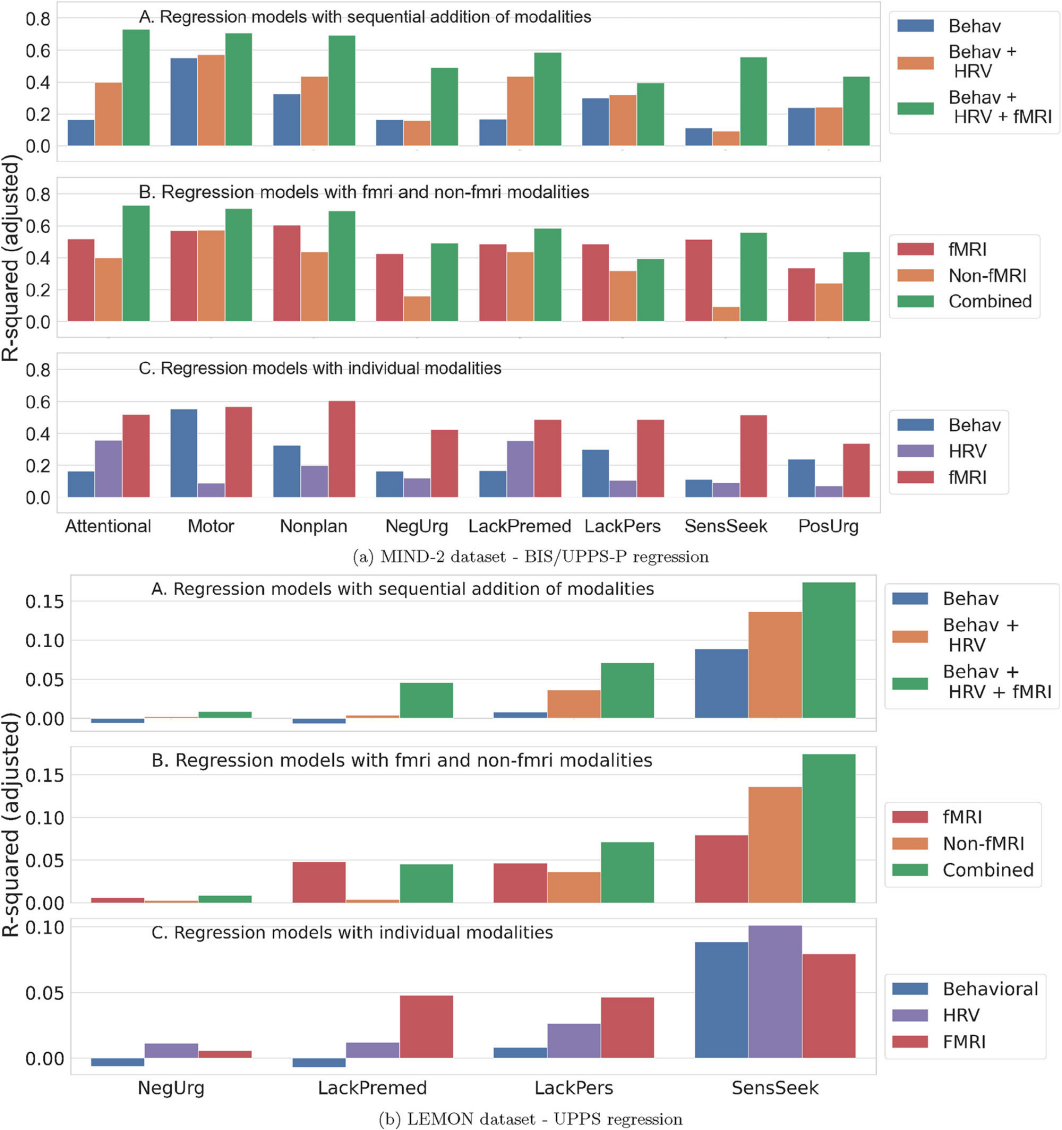
Regression model evaluation for different impulsivity dimensions. In each subfigure (MIND-2 dataset (**a**) and the LEMON dataset (**b**)), the top panel shows the regression model with increasing modalities, the middle panel shows regression model results comparing fMRI and non-fMRI modalities, and the bottom panel shows regression results for unimodal models. Modalities complement each other, and increasing modalities led to better r-squared metrics for regression models. Though fMRI provided the strongest representation of impulsivity, they were still complemented by the behavioral and HRV features.

We evaluated regression models for each impulsivity dimension in the MIND-2 and LEMON datasets and present the results obtained in Fig. 2. Three sets of experiments were conducted to gain insight into the relation of modalities to impulsivity modeling. First, we evaluated regression models with an increasing number of modalities in order of increasing complexity (behavioral, physiological, and neurobiological). The multimodal model with all modalities yielded the best r-squared values (adjusted) across impulsivity dimensions and dataset. Non-fMRI modalities still complemented the fMRI modality to increase the r-squared values of an only-fMRI model. Finally, the fMRI modality most commonly provided the highest r-squared across impulsivity dimensions and datasets in unimodal regression models.

As the features from the objective measurements showed an association with impulsivity, we also evaluated the predictive power of the features from these objective measurements to predict the impulsivity of an unseen participant. The prediction model based on a linear support vector machine and with behavioral (error rates and response times), physiological (HRV), and neurobiological (seed-to-ROI connectivity) features as input was evaluated with leave-one-participant-out cross-validation in the MIND-2 dataset (because the number of participants was small) and five-fold cross-validation in the LEMON dataset. Age and gender were added as predictors to account for possible age and gender-associated differences in functional connectivity [51, 52]. The results from the prediction model are given in Table 3. The predicted lack of premeditation, attentional, and nonplanning impulsivity significantly correlated with the self-reported impulsivity in the MIND-2 dataset. The predictions with and without the demographic features are shown in Supplementary Table S5. Adding the demographic features improved prediction specifically for the LEMON dataset where participants have diverse age groups (age range from 20 to 80 years compared to the 18 to 35 years in the MIND-2 dataset). Across the MIND-2 dataset and LEMON dataset, the most important features for prediction were IMT error rate, DMT response time, and TAP-I error rate in the behavioral modality; pNN20, SD1/SD2, and SDRR in the physiological modality; and ACC’s connectivity to the medial prefrontal cortex, Vermis 1, 2, and supramarginal gyrus in the neurobiological modality (Supplementary Section S6).

**Table 3:** Prediction of impulsivity in the held-out participants using multimodal behavioral, physiological, and neurobiological measurements.

	MIND-2 Dataset		LEMON Dataset	
	correlation	*p*-value	correlation	*p*-value
Negative Urgency	0.29	0.23	**−**0.01	0.88
Lack of Premeditation	**0.50**	**0.03**	**−0.20**	**0.01**
Lack of Perseverance	0.13	0.58	**0.19**	**0.02**
Sensation Seeking	0.16	0.51	**0.48**	**3** × **10**^−11^
Positive Urgency	**−**0.07	0.79		
Attentional	**0.79**	**7** × **10**^−5^		
Motor	0.35	0.14		
Nonplanning	**0.78**	**9** × **10**^−5^		

Predictions that correlated significantly with the actual values are highlighted.

## Discussion

Higher impulsivity is implicated in several health conditions. Objective impulsivity assessments could assist in the diagnosis and intervention monitoring of these health conditions. In this work, we investigated objective correlates of impulsivity in multimodal measurements comprising behavioral tests, HRV, and fMRI.

### Impulsivity (likely) manifests in multiple body systems

Correlates of impulsivity dimensions were found across the modalities in both the participants with mood disorders and healthy participants (Table 1). For instance, the lack of perseverance had correlates in the behavioral tests and HRV for both the MIND-2 and LEMON datasets. Even within a single impulsivity dimension, the correlates were usually obtained in multiple modalities. Regression models with multiple modalities showed that different modalities are complementary in representing the underlying impulsivity of the participants (Fig. 2). Collectively, these results indicate that the signature of impulsivity is imparted to various body systems through complementary mechanisms. If we aim to assess impulsivity accurately using objective measurements, employing multiple modalities might be required.

### Comparison to previous work on multimodal impulsivity assessment

In the only previous work that investigated multimodal impulsivity assessment [30], with a sample size similar to our MIND-2 dataset, the adjusted r-squared for impulsivity dimensions was reported to be 0.01 (minimum) to 0.30 (maximum). The authors used various mobile sensing-based features for impulsivity modeling, which provides a behavioral representation of individuals. In contrast, we obtained a higher adjusted r-squared in the MIND-2 dataset. Representation of the physiological and neurobiological characteristics, in addition to the behavioral data, helped get a more accurate representation of impulsivity.

### Impulsivity in the clinical and healthy population

Though the impulsivity regression model had a higher r-squared in the MIND-2 dataset - as high as 0.73 (*p* < 0.001) for attentional impulsivity, the r-squared was low for the LEMON dataset. The highest r-squared obtained was 0.17 (*p* < 0.001) for sensation seeking in the LEMON dataset despite using all behavioral, physiological, and neurobiological modalities. The LEMON dataset represented a wider age group (20–80 years) compared to MIND-2 (18–35 years). Even in a sub-sample of the LEMON dataset that is age and gender-matched with MIND-2 (and thus the same dataset size), the r-squared values for impulsivity modeling were lower compared to MIND-2 dataset (Supplementary Fig. S3, reported for an average of 100 random samplings, show maximum r-squared of 0.43 for sensation seeking in the LEMON dataset compared to the r-squared of 0.73 for attentional impulsivity in the MIND-2 dataset). The association between impulsivity dimensions was different among the mood disorder participants, represented by the MIND-2 dataset, and healthy participants, represented by the LEMON dataset (Supplementary Fig. S1). However, the distribution of the impulsivity scores across healthy and mood disorder participants was comparable (Supplementary Fig. S2). The higher r-squared for mood disorder participants could indicate different impulsivity mechanisms in this population that are more amenable to objective assessment with the employed behavioral, physiological, and neurobiological measurements. The association of sensation seeking with other impulsivity dimensions indicates different impulsivity mechanisms across clinical and healthy populations (Supplementary Fig. S1). In the clinical population, the sensation-seeking dimension is associated strongly with positive and negative urgency, indicating higher sensation-seeking behavior when under stress or excitation. In contrast, sensation seeking is associated with a lack of premeditation in the healthy participant cohort, i.e., the LEMON dataset. Higher sensation-seeking behavior was likely based on a lack of premeditation about the consequences or harms of the sought behavior. Future work on larger sample sizes across populations could better elucidate differences in impulsivity mechanisms of different population groups.

### Towards free-living/in-the-wild impulsivity monitoring

As impulsive actions have severe consequences on health behaviors, passively monitoring impulsivity in free living would be ideal to enable timely interventions. Behavioral and physiological measurements could enable such in-the-wild impulsivity monitoring, which is easier to deploy. Behavioral measurements could be deployed as routine games on smartphones and smartwatches. Continuous physiological measurements such as HRV are already possible with today’s wearables. However, our results show that though behavioral and physiological measurements can represent impulsivity variations across individuals, fMRI provides the strongest correlate (Fig. 2). fMRI had the highest r-squared in unimodal regression models. fMRI also improved regression model results further when added to the behavioral and physiological modalities in all cases (BIS and UPPS-P impulsivity dimension modeling and MIND-2 /LEMON dataset). fMRI’s strong association with impulsivity dimensions could be due to the direct pathway from brain connectivity to behavior [53]. Despite its relevance, fMRI is unsuitable for routine impulsivity assessment due to its associated costs. Brain connectivity measurement from other modalities in wearable form-factor, such as electroencephalogram (EEG), could still be prohibitive for deployment in free living. Thus, future work could consider several directions to improve impulsivity modeling with physiological and behavioral measurements alone. Some of these directions are new behavioral test paradigms, novel physiological features of impulsivity guided by the biology of impulsivity, and control of confounding factors that could influence behavioral and physiological measurements. The search for generalizable bio-behavioral markers of impulsivity should be expanded in larger studies and validated prospectively. This work’s MIND-2 study was relatively smaller and consisted of a homogeneous group. For example, the patients were enrolled from a single center and represented a small age group only (18 to 35 years). Given the encouraging findings on the possibility of obtaining behavioral, physiological, and neurobiological correlates of impulsivity, a larger study with diverse patient groups could shed light on the generalizability of these findings in the mood disorder population.

### Limitations of current work

The number of participants in the MIND-2 dataset was small. Thus, if the findings reported in this work generalize to a larger clinical population need to be investigated in future work. Apart from the small sample size in the MIND-2 study, other limitations of the current work need to be addressed in future studies. Though behavioral tests are commonly employed to assess impulsivity differences across participants [24], some studies have pointed out that behavioral test performance could be impacted by confounding factors such as intelligence (e.g., assessed using IQ scores) [54, 55]. Future studies should aim to capture and account for different confounders to represent impulsivity better. For the physiological measurements, we considered only the PPG signal. ECG signals could provide a more direct and reliable approach to assess HRV changes under different participant conditions [56]. The difference between PPG and ECG signal-based HRV calculation in the context of impulsivity assessment needs to be systematically evaluated. Finally, we relied only on resting-state fMRI signals in our current analysis. Though task-based fMRI was available for a subset of participants in the MIND-2 study, such task-based fMRI was not collected as part of the LEMON dataset. Future studies should investigate if better neurobiological correlates could be obtained with task-based fMRI measurements. Similarly, brain regions beyond ACC should be explored in the search for neurobiological impulsivity correlates. Larger studies could afford a broader search without a high risk of false discovery. With a better understanding of impulsivity mechanisms across clinical conditions, future studies should also investigate if a joint model of impulsivity that harmonizes impulsivity mechanisms across clinical conditions could be developed. Such joint models of impulsivity would better match the need for robust and scalable clinical monitoring.

## Supplementary information

Supplementary Materials: Multimodal Objective Assessment of Impulsivity in Healthy and Mood Disorder Participants

## Data Availability

The LEMON dataset used for the analysis in this work is available from the LEMON project website at https://fcon_1000.projects.nitrc.org/indi/retro/MPI_LEMON.html. We have also made the intermediate features and codes used for the analysis in a GitHub repository at https://github.com/lbishal/multimodal_impulsivity. The data and features from the MIND-2 dataset are not made publicly available due to the privacy-sensitive nature of the measurements for the clinical population. Corresponding author Bishal Lamichhane can be contacted at bishal.lamichhane@rice.edu for any data-related queries.
