# Supplementary material for: Multimodal objective assessment of impulsivity in healthy and mood disorder participants
Source: NPP Digit Psychiatry Neurosci. 2025 Feb 14;3:6. doi: 10.1038/s44277-025-00026-z (PMC12624883; doi:10.1038/s44277-025-00026-z)
Supplement: Supplementary file 1 — Supplementary Materials: Multimodal Objective Assessment of Impulsivity in Healthy and Mood Disorder Participants [file 44277_2025_26_MOESM1_ESM.pdf]

Supplementary Materials: Multimodal Objective Assessment of Impulsivity in Healthy and Mood Disorder Participants

S1. MIND-2 Dataset

Our study for the MIND-2 dataset comprised 34 participants with mood disorders, primarily major depressive disorder and bipolar disorder. We obtained four impulsivity-related measurements from the participants: questionnaires, behavioral tests, heart rate variability (HRV), and fMRI (functional magnetic resonance imaging). However, all participants did not have all four measurements due to participants opting out of some measurement sessions and technical issues during measurements. We obtained the correlation between impulsivity dimensions assessed from the questionnaire and objective modality-based impulsivity markers in the participant subset where both the questionnaire and the objective modality were available. Nineteen participants had all four measurement modalities available for multimodal impulsivity modeling analysis. The demographics of all relevant participant subsets are reported in Table S1. Statistics about the LEMON dataset can be obtained from [1].

TABLE S1
Participants' demographics for different subsets of MIND-2 dataset considered in our analysis. Since all participants did not have all four impulsivity-related measurements, we constructed the largest subsets that supported correlation analysis between measurement pairs and multimodal analysis. Participants were primarily diagnosed with either bipolar disorder, major depressive disorder (MDD), panic disorder, or post-traumatic stress disorder (PTSD)

Subset	Age	Gender	Diagnosis
All (N=34)	27.3 ± 5.2	15 M<br>19 F	21 MDD<br>11 Bipolar<br>1 PTSD<br>1 Panic Disorder
Impulsivity Questionnaire - Flanker correlation (N=29)	27.3 ± 5.3	14 M<br>15 F	18 MDD<br>9 Bipolar<br>1 PTSD<br>1 Panic Disorder
Impulsivity Questionnaire - IMT/DMT correlation (N=25)	26.5 ± 5.5	11 M<br>14 F	17 MDD<br>7 Bipolar<br>1 PTSD
Impulsivity Questionnaire - HRV correlation (N=28)	27.5 ± 5.5	14 M<br>14 F	19 MDD<br>7 Bipolar<br>1 PTSD<br>1 Panic Disorder
Impulsivity Questionnaire - fMRI correlation (N=31)	27.4 ± 5.3	13 M<br>18 F	19 MDD<br>10 Bipolar<br>1 PTSD<br>1 Panic Disorder
Multimodal impulsivity modeling/prediction (N=19)	26.4 ± 5.9	9 M<br>10 F	13 MDD<br>5 Bipolar<br>1 PTSD

S2. Correlation Between Impulsivity Dimension

We analyzed the association between impulsivity dimensions of the UPPS impulsivity (urgency, lack of premeditation, lack of perseverance, and sensation seeking) in the LEMON dataset and the impulsivity dimensions of the UPPS-P (UPPS dimensions along with the additional positive urgency dimension) and BIS-11 (Barratt Impulsiveness Scale) in the MIND-2 dataset. The results obtained are shown in Figure S1.

Differing associations between the dimensions (which pair of the dimensions have significant correlations and what is the correlation coefficient) are observed in the two datasets, with the LEMON dataset representing the healthy population and the MIND-2 dataset representing the mood disorder population. The UPPS impulsivity dimensions are available in both the MIND-2 and LEMON datasets. The distribution of the UPPS scores in the two datasets is shown in Figure S2. The spread of impulsivity scores across participants in the two datasets is similar except for the negative urgency. The MIND-2 dataset participants reported higher negative urgency than the LEMON dataset participants.

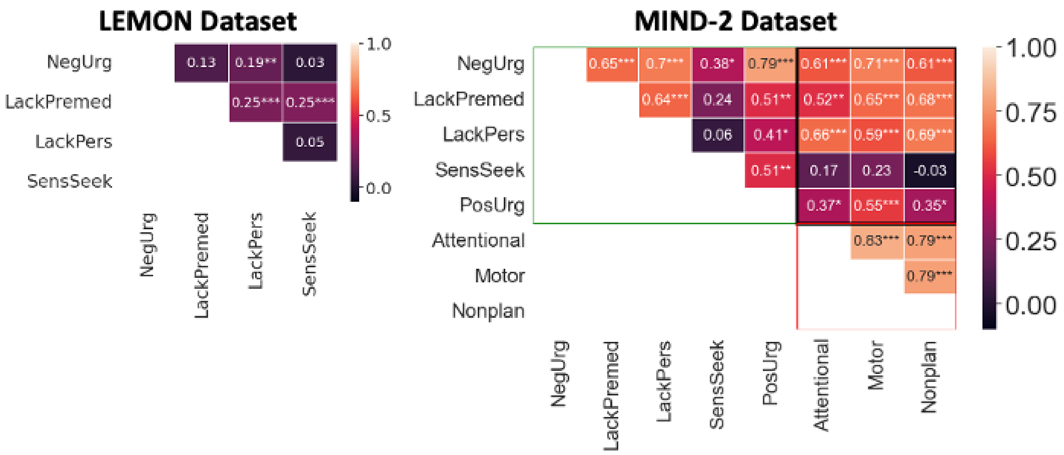

Fig. S1. Correlation between BIS and UPPS-P impulsivity dimensions in the MIND-2 dataset (top) and between the UPPS impulsivity dimensions in the LEMON dataset (bottom). BIS dimensions are attentional, motor, and nonplanning. UPPS-P dimensions are negative urgency, lack of premeditation, lack of perseverance, sensation seeking, and positive urgency. The MIND-2 dataset consisted of participants with mood disorders, and the LEMON dataset represents the healthy population. The two datasets show differing associations between impulsivity dimensions and correlation strength.

S3. fMRI Pre-processing and Quality Analysis in the MIND-2 and LEMON datasets

The fMRI scans for the MIND-2 dataset were obtained with a 3T Seimens Prisma Fit machine. A structural scan was obtained with a repetition time of 2.3 seconds, and the functional scans were obtained with a repetition time of 1 second. A total of 375 functional scans were obtained in the resting state. The voxel size for the structural scan was $1\times1\times1mm^3$, and that for the functional scan was $2.2\times2.2\times2.2mm^3$. The fMRI data was processed using the CONN toolbox [2]. The preprocessing steps included functional realignment and unwarp, slice-timing correction, outlier identification, direct segmentation and normalization, and functional smoothing as specified in the default pipeline [2]. The fMRI scans in the LEMON dataset were obtained with a 3T Siemens Verio machine [3]. The structural scan was obtained with a repetition time of five seconds, and the functional scans were obtained with a repetition time of 1.4 seconds. The voxel size for the structural scan was $1 \times 1 \times 1\,mm^3$, and that for the functional scan was $2.3 \times 2.3 \times 2.3\,mm^3$. The fMRI data was preprocessed with a pipeline implemented in Nipype [4] as provided in [3]. We used the preprocessed data to obtain functional connectivity between brain regions.

The quality of fMRI data was assessed in the LEMON dataset using the mean and maximum displacement metrics and qualitative analysis of the coregistrations of the functional and structural scans [1]. We analyzed the fMRI data quality in the MIND-2 dataset using the same metrics of mean and maximum displacement as in the LEMON dataset. We also performed qualitative analysis with visual inspections of normalized and segmented structural and functional images generated by the CONN toolbox. We ascertained that functional connectivity distribution is reasonably centered and similar across participants. Both the LEMON and MIND-2 datasets had high fMRI data quality. For example, the mean displacement in the LEMON dataset was 0.18 mm (SD=0.08 mm) [3], and that in the MIND-2 dataset was 0.20 mm (SD=0.08 mm). Similarly,

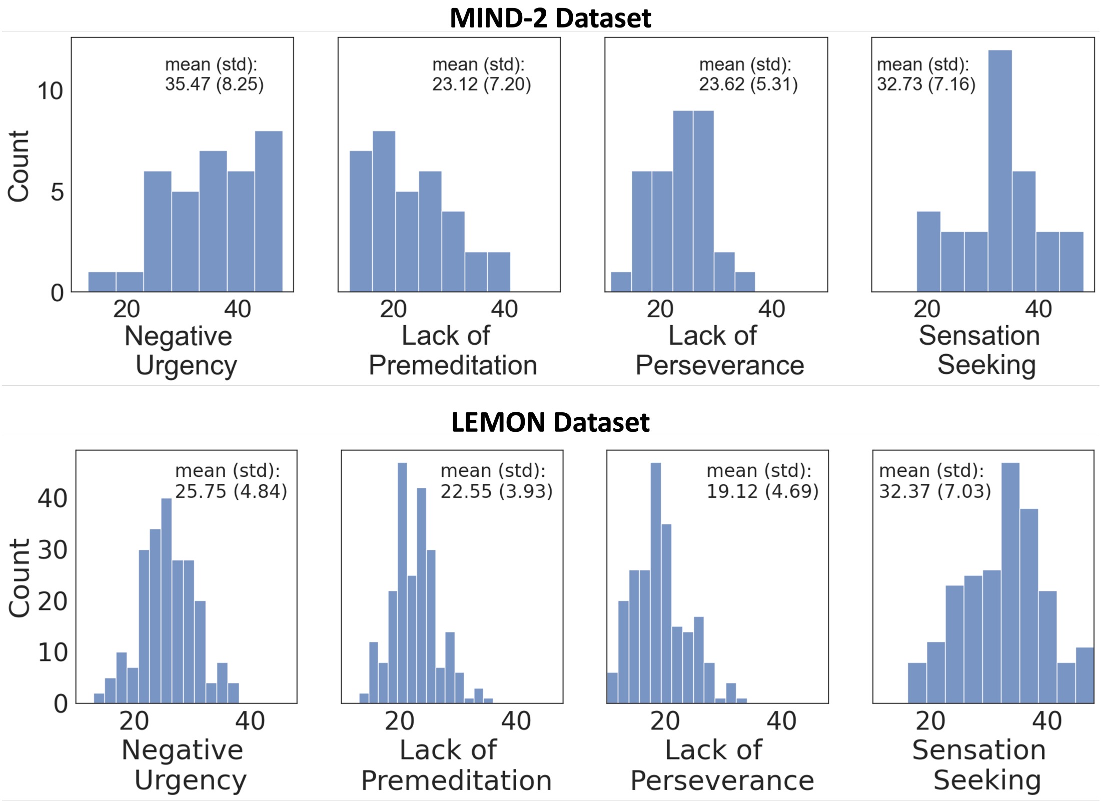

Fig. S2. Distribution of UPPS scores across MIND-2 and LEMON dataset. Both datasets have a similar spread of scores across participants except for the negative urgency. The MIND-2 dataset participants reported higher negative urgency than the LEMON dataset participants.

the maximum displacement being smaller than a voxel size was 89% in the LEMON dataset and 86% in the MIND-2 dataset.

S4. Quality Analysis of Behavioral Tests

We used the Flanker and the IMT/DMT (Immediate memory test/Delayed memory test) test as behavioral tasks to assess participant's impulsivity in the MIND-2 dataset. The quality of the behavioral test deployments and participant's responses were evaluated with the distribution of the response time and error rates for different kinds of trials in these tests. For example, the distribution of response time and average error rates for the congruent and incongruent trials of the Flanker test is shown in Figure S3. The congruent trials had lower response times and error rates, indicating correct deployment. In the IMT/DMT tests, the frequency of commission errors was substantially higher than the filler and distractor errors, as is to be expected [5].

S5. Correlation of Features from Objective Modalities with Impulsivity Scores

We obtained the Pearson's correlation coefficient between features obtained from the objective measurement modalities and impulsivity dimensions of BIS and UPPS-P/UPPS. The correlations with $p < 0.10$ is shown in Tables S2,S3,S4 to highlight only the possibly important correlations. Though the significance is conventionally set at $p < 0.05$, which is met by only a few correlation coefficients, especially after considering multiple comparisons, weaker correlations could still be relevant for downstream regression and prediction tasks. The scatterplot showing some example associations between behavioral/physiological measures and impulsivity is shown in Figure S4.

TABLE S2

Correlates of BIS impulsivity in the behavioral test, HRV, and fMRI-based features in the MIND-2 dataset. Pearson's correlation coefficient was computed, and only the correlations with p-value<0.10 are shown for decluttered results.

**Behavioral Tests**	**Attentional**	**Motor**	**Nonplanning**
Flanker - Error rate		0.35 (p=0.06)	0.41 (p=0.03)
Flanker - Response Time			
IMT Error Rate			
IMT Response Time			
DMT Error Rate		0.49 (p=0.01)	0.36 (p=0.07)
DMT Response Time			
**HRV**	**Attentional**	**Motor**	**Nonplanning**
mhr	-0.37 (p=0.05)	-0.36 (p=0.06)	
sdrr			
rmssd	0.35 (p=0.07)		
pnn50	0.45 (p=0.02)	0.33 (p=0.09)	
pnn20	0.52 (p<0.001)	0.40 (p=0.04)	
sd1	0.53 (p<0.001)		0.45 (p=0.01)
sd2	0.53 (p<0.001)		0.47 (p=0.01)
sd1/sd2			
**ACC-ROI connectivity**	**Attentional**	**Motor**	**Nonplanning**
PFC R	0.62 (p<0.001)		0.67 (p<0.001)
MidFG R	0.63 (p<0.001)		0.65 (p<0.001)
Cereb1 L			0.64 (p<0.001)
Medial		-0.70 (p<0.001)	
ICC R		-0.61 (p<0.001)	
Hippocampus R			-0.63 (p<0.001)

TABLE S3

Correlates of UPPS-P impulsivity in the behavioral test, HRV, and fMRI-based features in the MIND-2 dataset. Pearson's correlation coefficient was computed, and only the correlations with p-value<0.10 are shown for decluttered results.

**Behavioral Tests**	**Negative Urgency**	**Lack of Premeditation**	**Lack of Perseverance**	**Sensation Seeking**	**Positive Urgency**
Flanker - Error rate		0.47 (p=0.01)			0.32 (p=0.09)
Flanker - Response Time					
IMT Error Rate					
IMT Response Time					0.38 (p=0.06)
DMT Error Rate					
DMT Response Time	-0.36 (p=0.07)		-0.37 (p=0.07)		-0.34 (p=0.09)
**HRV**	**Negative Urgency**	**Lack of Premeditation**	**Lack of Perseverance**	**Sensation Seeking**	**Positive Urgency**
mhr		-0.35 (p=0.07)			
sdrr					
rmssd		0.45 (p=0.02)			
pnn50	0.37 (p=0.06)	0.48 (p=0.01)	0.33 (p=0.09)		0.37 (p=0.05)
pnn20		0.48 (p=0.01)			
sd1		0.42 (p=0.02)			
sd2		0.44 (p=0.02)			
sd1/sd2					
**ACC-ROI connectivity**	**Negative Urgency**	**Lack of Premeditation**	**Lack of Perseverance**	**Sensation Seeking**	**Positive Urgency**
Cereb1 L		0.61 (p<0.001)			

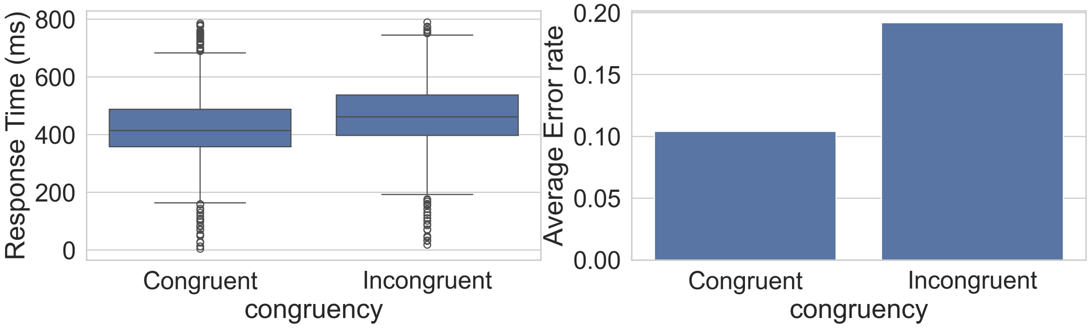

Fig. S3. Response time for the congruent and incongruent trials in the Flanker test (left) and the average error rate for these trials (right). As expected, the incongruent trials had higher response times and higher error rates than the congruent trials.

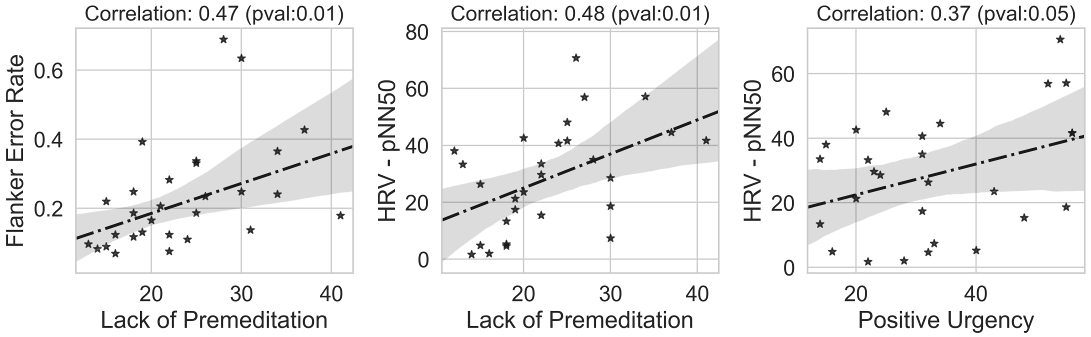

Fig. S4. Scatterplot showing example association between behavioral and physiological features with impulsivity in the MIND-2 dataset. The first plot shows the association between the error rate from the Flanker test and the lack of premeditation. The second plot shows the association between pNN50, a heart rate variability (HRV) feature computed from the photoplethysmography (PPG) signal, and the lack of premeditation. The third plot shows the association of the same save HRV feature of pNN50 with positive urgency, another impulsivity dimension captured by the UPPS-P questionnaire.

S6. Effect of Demographic Features in Impulsivity Prediction

We evaluated multimodal impulsivity prediction models with leave-one-participant-out cross-validation using behavioral, physiological, and neurobiological features. We added the demographic features of age and gender to help models better contextualize age and gender-associated feature changes, e.g., in the fMRI-based neurobiological measurements [6], [7]. We conducted experiments to assess the impact of adding demographic features in the impulsivity prediction model with behavioral, physiological, and neurobiological features as input. The results obtained for the prediction model with and without the demographic features are shown in Table S5. The demographic features improved prediction, specifically for the LEMON dataset obtained from participants representing diverse demographics (age range from 20 to 80 years, compared to the 18 to 35 years in the MIND-2 dataset). In the impulsivity prediction model, we assessed the feature importance for each cross-validation loop based on the learned weights assigned to the features in the linear support vector machine model. The most commonly selected important features (among the top five features) in the behavioral modality for the MIND-2 dataset were IMT error rate, DMT response time, and TAP-I error rate in the LEMON dataset. In the physiological modality, the most important features were pNN20, SD1/SD2, and SDRR for the MIND-2 dataset and pNN20 and SD1/SD2 for the LEMON dataset. In the neurobiological modality, the connectivity of ACC to the medial prefrontal cortex and Vermis 1,2 were most commonly selected in the MIND-2 dataset and to the supramarginal gyrus in the LEMON dataset.

TABLE S4
CORRELATES OF UPPS IMPULSIVITY IN THE BEHAVIORAL TEST, HRV, AND FMRI-BASED FEATURES IN THE LEMON DATASET. PEARSON'S CORRELATION COEFFICIENT WAS COMPUTED, AND ONLY THE CORRELATIONS WITH P-VALUE<0.10 ARE SHOWN FOR DECLUTTERED RESULTS.

**Behavioral Tests**	**Negative Urgency**	**Lack of Premeditation**	**Lack of Perseverance**	**Sensation Seeking**
TAP-I Error Rate				
TAP-I Response Time			-0.13 (p=0.05)	-0.40 (p<0.001)
**HRV**	**Negative Urgency**	**Lack of Premeditation**	**Lack of Perseverance**	**Sensation Seeking**
mhr				
sdrr				
rmssd				0.13 (p=0.074)
pnn50				-0.16 (p=0.037)
pnn20				0.28 (p<0.001)
sd1				0.14 (p=0.07)
sd2				
sd1/sd2	-0.16 (p=0.038)	-0.21 (p=0.005)	-0.15 (p=0.048)	
**ACC-ROI connectivity**	**Negative Urgency**	**Lack of Premeditation**	**Lack of Perseverance**	**Sensation Seeking**
Thalamus L				-0.26 (p<0.001)

TABLE S5
PREDICTION OF IMPULSIVITY IN LEAVE-ONE-PARTICIPANT-OUT CROSS-VALIDATION USING MULTIMODAL BEHAVIORAL, PHYSIOLOGICAL, AND NEUROBIOLOGICAL MEASUREMENTS. PREDICTIONS THAT CORRELATED SIGNIFICANTLY WITH THE REPORTED IMPULSIVITY ARE HIGHLIGHTED.

	**MIND-2 Dataset**				**LEMON Dataset**			
	**With Demographics**		**Without Demographics**		**With Demographics**		**Without Demographics**	
	correlation	p-value	correlation	p-value	correlation	p-value	correlation	p-value
Negative Urgency	0.29	0.23	0.30	0.21	-0.01	0.88	-0.06	0.39
Lack of Premeditation	**0.50**	**0.03**	**0.52**	**0.02**	**-0.20**	**0.01**	**-0.18**	**0.01**
Lack of Perseverance	0.13	0.58	0.12	0.61	**0.19**	**0.02**	0.03	0.62
Sensation Seeking	0.16	0.51	0.21	0.38	**0.48**	$\mathbf{3 \times 10^{-11}}$	**0.29**	$\mathbf{1.4 \times 10^{-4}}$
Positive Urgency	-0.07	0.79	-0.14	0.56				
Attentional	**0.79**	$\mathbf{7 \times 10^{-5}}$	**0.81**	$\mathbf{2 \times 10^{-5}}$				
Motor	0.35	0.14	0.38	0.11				
Nonplanning	**0.78**	$\mathbf{9 \times 10^{-5}}$	**0.77**	$\mathbf{1 \times 10^{-4}}$				

S7. AGE AND GENDER-MATCHED IMPULSIVITY REGRESSION MODELS IN LEMON DATASET

We found that the regression model of impulsivity provided a higher r-squared for the MIND-2 dataset (r-squared of up to 0.73 for attentional impulsivity) compared to the LEMON dataset (r-squared of 0.17 for sensation seeking) as shown in Figure 2 of the manuscript. The participants in the MIND-2 dataset and the LEMON datasets have different demographics. Specifically, while the MIND-2 dataset had only younger participants (18 to 35 years), the LEMON dataset consisted of both young and older participants (20 to 80 years). Similarly, the LEMON dataset had a higher representation of male participants (gender ratio of 1.76) while the MIND-2 dataset had a more balanced composition across genders in comparison (gender ratio of 1.26). To compare the regression models of impulsivity in the age and gender-matched MIND-2 and LEMON datasets, we sub-sampled the LEMON dataset (the larger dataset) to match the age and gender distribution of the MIND-2 dataset (the smaller dataset). In particular, for each participant in the MIND-2 dataset, we randomly selected a LEMON dataset participant with the same age and gender to be included in the analysis subset. We evaluated the ordinary linear regression model for the sub-sampled LEMON dataset with behavioral, physiological, and neurobiological features as input. The regression modeling approach was the same as described in the *Methods/Multimodal Regression and Prediction Model* section in the manuscript. Due to the random selection, we repeated the procedure 100 times and

report the obtained regression results in Figure S5. Though the r-squared were better than those obtained with the entire dataset (e.g., r-squared of 0.43 for sensation seeking compared to 0.17, as shown in Figure 2 in the manuscript), the r-squared values were still less than those obtained with the MIND-2 dataset (Figure 2 in manuscript which showed r-squared of up to 0.73 for attentional impulsivity). The results confirm lower performance in impulsivity modeling with objective measurements for healthy participants. The MIND-2 dataset and thus the age and gender-matched LEMON subset for analysis only comprised the young population (age $<$ 35 years). To understand if there are any differences in impulsivity modeling for older and younger sub-samples of the LEMON dataset population, we pursued similar regression model analysis on the LEMON dataset participants who are older. To obtain a sub-sample of older participants, we sub-sampled the same number of participants as MIND-2, gender-matched, but age offset by 35 years. The results obtained are shown in Figure S6. The r-squared obtained with multimodal measurements is comparable to those obtained for the sub-sampling of younger participants (Figure S5). The results indicate that the impulsivity modeling is less accurate in the healthy participants compared to the clinical population of mood disorder participants, irrespective of the age group of the cohort.

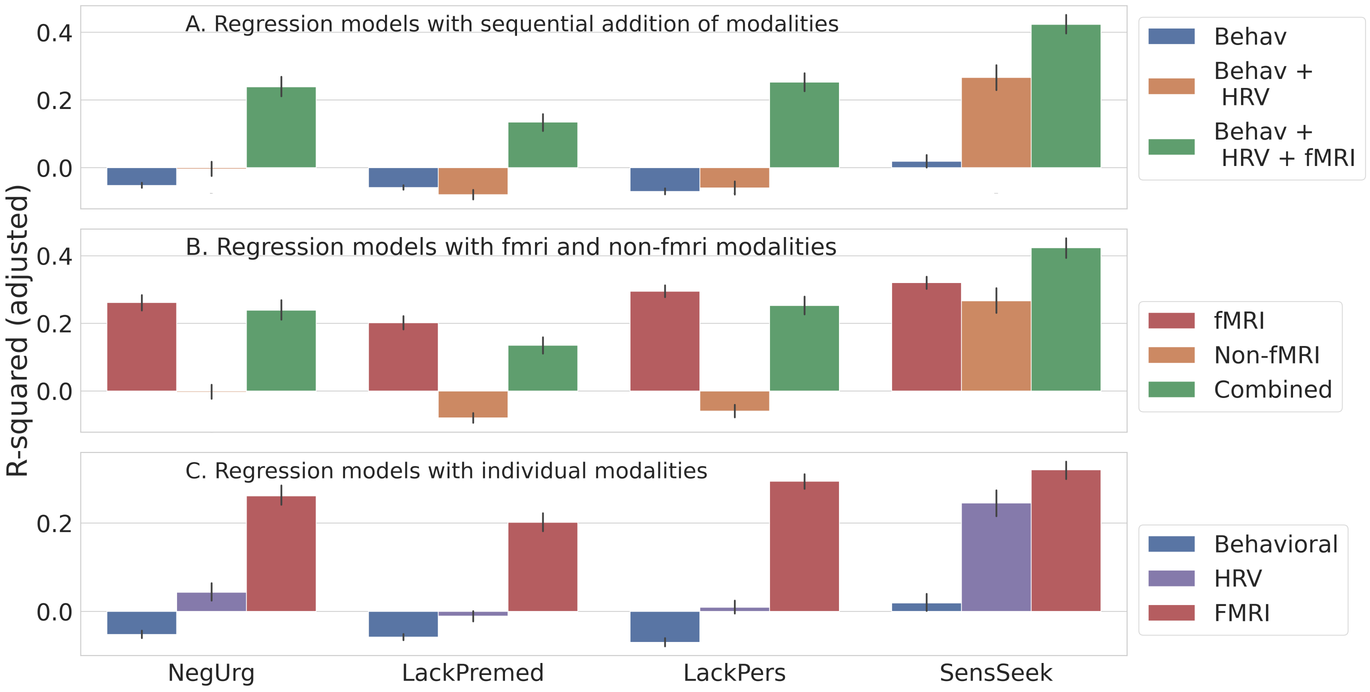

Fig. S5. Regression model evaluation for different impulsivity dimensions in the sub-sampled LEMON dataset such that the age and gender distribution is matched with the MIND-2 dataset. In each subfigure, the top panel shows the regression model with increasing modalities, the middle panel shows regression model results comparing fMRI and non-fMRI modalities, and the bottom panel shows regression results for unimodal models. Modalities complement each other, and increasing modalities led to better r-squared metrics for regression models. Though fMRI provided the strongest representation of impulsivity, they were still complemented by the behavioral and HRV features. The average results for 100 runs are shown.

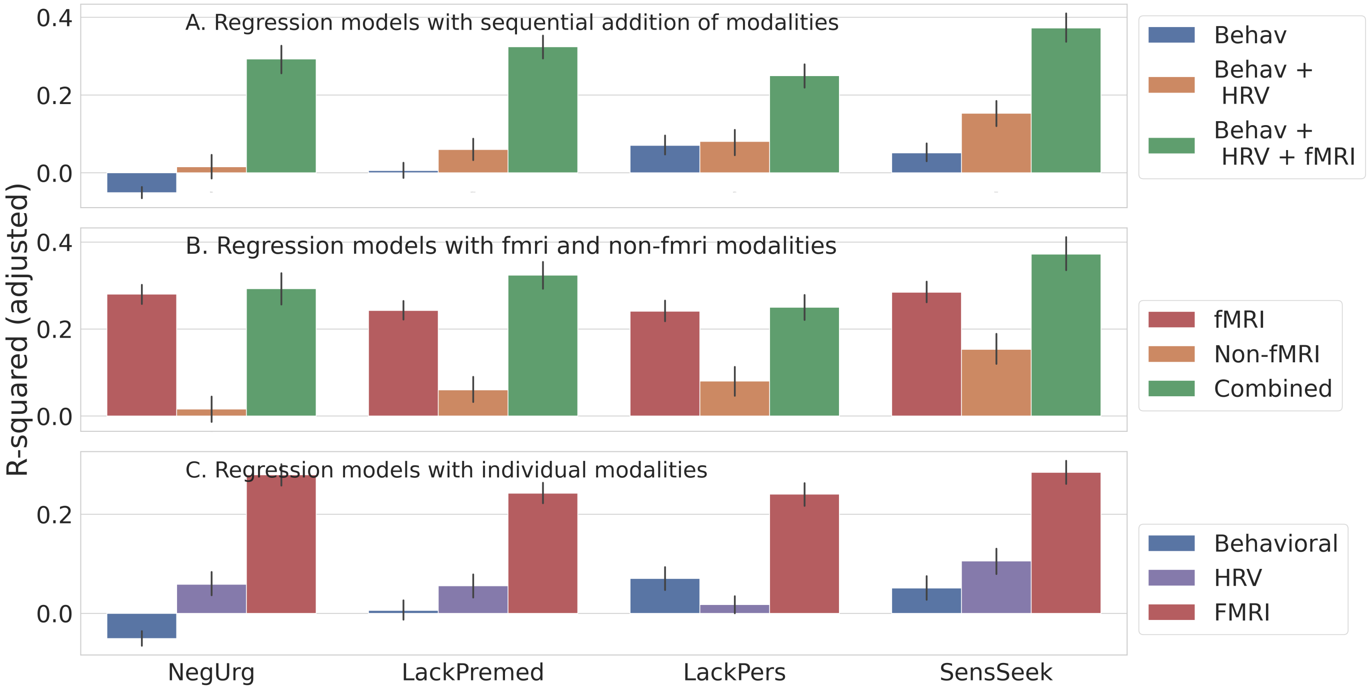

Fig. S6. Regression model evaluation for different impulsivity dimensions in the sub-sampled LEMON dataset (older age group) such that gender distribution is matched with the MIND-2 dataset. In each subfigure, the top panel shows the regression model with increasing modalities, the middle panel shows regression model results comparing fMRI and non-fMRI modalities, and the bottom panel shows regression results for unimodal models. Modalities complement each other, and increasing modalities led to better r-squared metrics for regression models. Though fMRI provided the strongest representation of impulsivity, they were still complemented by the behavioral and HRV features. The average results for 100 runs are shown.

REFERENCES

[1] Anahit Babayan, Miray Erbey, Deniz Kumral, Janis D Reinelt, Andrea MF Reiter, Josefin Röbbig, H Lina Schaare, Marie Uhlig, Alfred Anwander, Pierre-Louis Bazin, et al. A mind-brain-body dataset of mri, eeg, cognition, emotion, and peripheral physiology in young and old adults. *Scientific data*, 6(1):1–21, 2019.

[2] Susan Whitfield-Gabrieli and Alfonso Nieto-Castanon. Conn: a functional connectivity toolbox for correlated and anticorrelated brain networks. *Brain connectivity*, 2(3):125–141, 2012.

[3] Natacha Mendes, Sabine Oligschläger, Mark E Lauckner, Johannes Golchert, Julia M Huntenburg, Marcel Falkiewicz, Melissa Ellamil, Sarah Krause, Blazej M Baczkowski, Roberto Cozatl, et al. A functional connectome phenotyping dataset including cognitive state and personality measures. *Scientific data*, 6(1):1–19, 2019.

[4] Krzysztof Gorgolewski, Christopher D Burns, Cindee Madison, Dav Clark, Yaroslav O Halchenko, Michael L Waskom, and Satrajit S Ghosh. Nipype: a flexible, lightweight and extensible neuroimaging data processing framework in python. *Frontiers in neuroinformatics*, 5:12318, 2011.

[5] Donald M Dougherty, Dawn M Marsh, and Charles W Mathias. Immediate and delayed memory tasks: a computerized behavioral measure of memory, attention, and impulsivity. *Behavior research methods, instruments, & computers*, 34(3):391–398, 2002.

[6] Richard F Betzel, Lisa Byrge, Ye He, Joaquín Goñi, Xi-Nian Zuo, and Olaf Sporns. Changes in structural and functional connectivity among resting-state networks across the human lifespan. *Neuroimage*, 102:345–357, 2014.

[7] Linda Geerligs, Kamen A Tsvetanov, and Richard N Henson. Challenges in measuring individual differences in functional connectivity using fmri: the case of healthy aging. *Human brain mapping*, 38(8):4125–4156, 2017.
